# Use of Continuous Electronic Fetal Monitoring in a Preterm Fetus: Clinical Dilemmas and Recommendations for Practice

**DOI:** 10.1155/2011/848794

**Published:** 2011-09-13

**Authors:** Karolina Afors, Edwin Chandraharan

**Affiliations:** St. George's Healthcare NHS Trust, Blackshaw Road, London SW17 0QT, UK

## Abstract

The aim of intrapartum continuous electronic fetal monitoring using a cardiotocograph (CTG) is to identify a fetus exposed to intrapartum hypoxic insults so that timely and appropriate action could be instituted to improve perinatal outcome. Features observed on a CTG trace reflect the functioning of somatic and autonomic nervous systems and the fetal response to hypoxic or mechanical insults during labour. Although, National Guidelines on electronic fetal monitoring exist for term fetuses, there is paucity of recommendations based on scientific evidence for monitoring preterm fetuses during labour. Lack of evidence-based recommendations may pose a clinical dilemma as preterm births account for nearly 8% (1 in 13) live births in England and Wales. 93% of these preterm births occur after 28 weeks, 6% between 22–27 weeks, and 1% before 22 weeks. Physiological control of fetal heart rate and the resultant features observed on the CTG trace differs in the preterm fetus as compared to a fetus at term making interpretation difficult. This review describes the features of normal fetal heart rate patterns at different gestations and the physiological responses of a preterm fetus compared to a fetus at term. We have proposed an algorithm “ACUTE” to aid management.

## 1. CTG Monitoring of a Preterm Fetus: The Current Status

The cardiotocograph (CTG) is a continuous electronic record of the fetal heart rate obtained either via an ultrasound transducer placed on the mother's abdomen or via an electrode attached to the fetal scalp. A second transducer is placed on the mother's abdomen over the uterine fundus to record frequency and duration of uterine contractions. Both components are then traced simultaneously on a paper strip. Based on current scientific evidence, a CTG is not recommended in the UK as a method of routine fetal assessment of the preterm fetus (<37 weeks gestation) and currently no clinical practice guidelines on intrapartum monitoring of the preterm fetus exist in the UK The International Federation of Gynaecologists and Obstetricians (FIGO) guidelines for interpretation of intrapartum cardiotocogram distinguish 2 levels of abnormalities, suspicious and pathological, however, the gestation to which such criteria can be applied has not been specified. The American College of Obstetricians and Gynaecologists (ACOG) published a practice bulletin on intrapartum fetal heart rate monitoring in 2009. Within this guideline, the decision to monitor the preterm fetus remains vague with recommendations that each case requires discussion between obstetric and neonatal input, in addition to weighing up likelihood of severe morbidity of the preterm fetus (based on gestational age and fetal weight) and issues related to mode of delivery [1]. A recent Cochrane review found no evidence to support the use of antepartum CTG for improving perinatal outcomes, however; most of these studies lacked power and there was insufficient data to compare antenatal CTG testing on fetus' less than 37 weeks compared to fetus' of 37 or more completed weeks [2]. 

Due to the lack of research and evidence that exists on electronic fetal monitoring (EFM) of the preterm fetus the definition of a normal fetal heart pattern also presents a challenge. Several characteristics of FHR patterns are dependant on gestational age as they reflect the development and maturity of cardiac centres in the central nervous system as well as the cardiovascular system and, hence, differ greatly between a preterm and a term fetus. Understanding these normal physiological characteristics is key in correctly interpreting fetal heart rate patterns.

## 2. Factors That Affect Fetal Heart Rate during Labour

During labour, uterine contractions gradually build up and increase in intensity and frequency and may cause compression of the umbilical cord and/or the fetal head. These “mechanical compressions” may result in decelerations in fetal heart resulting in early and variable decelerations, respectively. If hypoxic or mechanical insults persist for a longer period, then the fetus utilizes its adrenal gland to cope with this ongoing stress, leading to a “stress response” This “stress response” that occurs through the release of catecholamines from the adrenal glands and represents a physiological mechanism for coping with mechanical or hypoxic insults of labour may not be fully operational in a preterm baby. This may also be the case when the normal physiological reserves of the fetus may be impaired (intra-uterine growth restriction, fetal infection). Inability of a preterm or growth restricted fetus to mount a required stress response may lead to maladaptive responses resulting in permanent hypoxic insult on the fetal brain occurring at a lower threshold than in the term fetus. Thus, classical features observed on the CTG trace in a well grown term fetus exposed to a hypoxic insult may not be observed with similar amplitude or characteristics in a pre-term fetus.

Fetal heart rate is regulated by the autonomic nervous system consisting of 2 branches; the parasympathetic and sympathetic branch which exerts opposing influences on the FHR. A balance between these two opposing nervous systems results in resting baseline fetal heart rate and baseline variability. During fetal development, the sympathetic nervous system that is responsible for survival (“fight or flight” response) develops much earlier than the parasympathetic nervous system (“rest and sleep”) that develops during the third trimester. Hence, a preterm fetus may have a higher baseline fetal heart rate with apparent reduction of baseline variability due to unopposed action of sympathetic nervous system.

### 2.1. Baroreceptors

The parasympathetic nervous system is activated by stimulation of baroreceptors situated in the carotid sinus or aortic arch secondary to increase in fetal systemic blood pressure, leading to a fall in heart rate mediated through the vagus nerve. This is illustrated by a deceleration on a CTG. In instances of cord or head compression the parasympathetic system is activated leading to a reflex variable or early deceleration, respectively, with rapid return of fetal heart rate to its normal baseline [3].

### 2.2. Chemoreceptors

Chemo-receptors are located peripherally within the aortic and carotid bodies and centrally in the medulla oblongata. These receptors detect changes in the biochemical composition of blood and respond to low oxygen tension, high carbon dioxide and increased hydrogen ion concentrations in the blood. In cases of utero-placental insufficiency, where carbon dioxide and hydrogen ion accumulate with resultant decrease in oxygen concentrations, the chemo-receptors are activated. This results in parasympathetic activation leading to a fall in heart rate, which is protracted and takes longer to recover to baseline rate. These types of decelerations are termed “late” decelerations and due to the accumulation of carbon dioxide and hydrogen ions are more suggestive of metabolic acidosis [3].

### 2.3. Somatic Nervous System

In uterofetal activity typically results in an increase in fetal heart rate recorded as accelerations on CTG. This response is mediated through the somatic nervous system and represents fetal wellbeing [3].

### 2.4. Fetal Adrenal Glands

**Figure 1:**
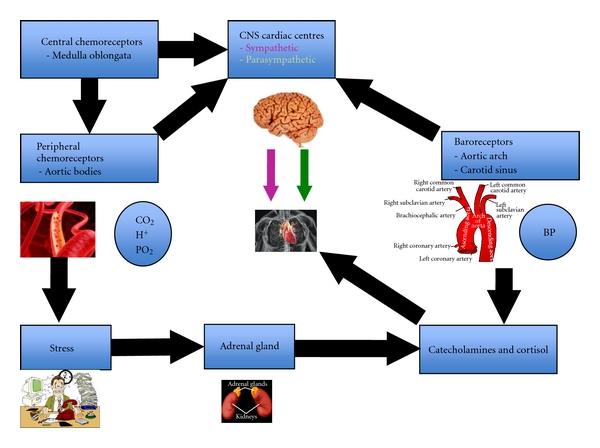
Pathophysiology of fetal heart rate changes.

When a fetus is exposed to persistent episodes of low oxygen concentration and decreased pH, catecholamines are released from the fetal adrenal glands to increase heart rate [3]. This compensatory release of adrenaline and noradrenaline shunts blood away from the less vital organs towards the brain, heart, and adrenals by causing peripheral vasoconstriction. This clinical scenario of decelerations, followed by loss of accelerations, subsequent rise in baseline heart rate and gradual loss of variability is typical of a gradually evolving hypoxia (Figure 1). 

## 3. Characteristics of Fetal Heart Rate in a Preterm Fetus

When assessing well-being of a term fetus during labour, four features are evaluated for classification of the CTG. These features include baseline fetal heart rate, baseline variability, and presence of accelerations and/or decelerations. According to National Institute of Health and Clinical Excellence (NICE) guidelines on electronic fetal monitoring in labour, these features, which are present in labour, are further categorized into reassuring and nonreassuring as outlined in Table 1 below.

Characteristics of antepartum and intrapartum fetal heart rate tracings differ in the preterm fetus as compared to a term fetus. Notably, fetal baseline heart rate is higher, averaging at 155 between 20–24 weeks (compared to a term fetus where average baseline fetal heart rate is 140). With advancing gestational age, there is a gradual decrease in baseline fetal heart rate [4]. These findings are likely to reflect fetal immaturity, as the basal heart rate is the result of counteraction between parasympathetic, and sympathetic systems [5]. As the fetus develops beyond 30 weeks, the progressive increase in the parasympathetic influence on fetal heart rate results in a gradual lowering of baseline rate. 

Fetal heart rate accelerations are also noted to change with advancing gestational age. Accelerations of fetal heart rate in association with fetal movements occur as a result of fetal somatic activity and are first apparent in the 2nd trimester. Before 30 weeks of gestational age, the frequency and amplitude of accelerations are reduced. Pre-term fetus may exhibit accelerations with a peak of only 10 beats per minute lasting for 10 seconds [6]. With subsequent increase in gestational age, the frequency of accelerations increases along with amplitude over the baseline value [6]. 

Fetal heart rate decelerations in the absence of uterine contractions often occur in the normal preterm fetus between 20 and 30 weeks gestation. As described by Sorokin et al. these decelerations have a lower depth and duration, but can be seen frequently on intrapartum CTG tracings [4]. Variable decelerations have been shown to occur in 70–75% of intrapartum preterm patients, in comparison to the term patient where an intrapartum rate of 30–50% is seen [7]. Several theories have been proposed as a potential explanation for this fetal heart rate pattern, notably decreased amount of amniotic fluid, reduced the Wharton jelly component in the cord of the preterm fetus and lack of development of the fetal myocardium and, therefore, the resultant reduced force of contraction. 

Baseline variability may be affected due to incomplete development of autonomic nervous system and subsequent interplay between parasympathetic and sympathetic systems. Variability may also be decreased secondary to the effect of fetal tachycardia present in preterm fetuses. Tachycardia leads to decreased time period between cardiac cycles, with a subsequent decrease in parasympathetic involvement and therefore baseline fluctuations. Reduction in fetal baseline variability in the preterm fetus has been described, however this has not been quantified. Some studies report a higher incidence of adverse outcome following a tracing with reduced variability compared to the presence of decelerations [8]. 

One of the hallmarks of fetal wellbeing is considered to be “cycling” of the fetal heart rate [3]. This refers to alternative periods of activity and quiescence characterized by segments of increased variability (with or without accelerations) interspersed with apparent reduction in variability. These are believed to reflect Rapid Eye Movement (REM) and non-REM sleep. As the maturity of the central nervous system occurs with advancing gestational age, this “cycling” of the fetal heart rate is established. Hence, in an extreme preterm infant, cycling may be absent and this may be due to functional immaturity of the central nervous system, rather than hypoxic insult.

## 4. Interpreting Intrapartum CTG at Different Gestations

### 4.1. 24–26 Weeks

Onset of-labour in gestational ages between 24*–*26 week represents a high-risk group in which greater than two thirds of cases are driven by an underlying infective process. Other possible factors that may contribute to onset of labour in this group include multiple gestations maternal risk factors such as increased maternal age, raised body mass index (BMI), or pregnancies conceived through in-vitro fertilization (IVF). At this gestation, there is a high risk of neonatal morbidity and mortality, and survival is dependant more on fetal weight and maturity rather than mode of delivery. Hence, continuous monitoring of the fetus during labour, with the view to recognizing features of suspected fetal compromise on CTG and instituting an operative intervention, should be considered with caution. The use of CTG monitoring in this group is contentious and each case should be considered individually with a plan of care agreed following discussion between the patient, obstetrician, and neonatologists. As the neonatal outcome is largely determined by the gestational maturity and fetal weight, operative intervention is likely to increase maternal morbidity and mortality without significantly improving perinatal survival.

**Practice Points**

Baseline fetal heart rate in this cohort of fetuses is likely to remain at the higher end of normal (between 150*–*160) due to the unopposed effect of the sympathetic nervous system. Although, the baseline heart rate is expected to be higher, any rate greater than 160 should be still considered to be tachycardic. Persistent tachycardia is likely to arise secondary to iatrogenic causes such as administration of tocolytics (terbutaline) [9]. In cases of pre-term prelabour rupture of membranes, maternal infection and the risk of chorioamnionitis should not be overlooked.

Baseline variability and cycling may be reduced at this gestation as a result of impaired development of the parasympathetic component of the autonomic nervous system. Medications such as pethidine, magnesium sulphate and even steroids have also been associated with reduced fetal heart rate variability. However, fetal heart rate variability is an important clinical indicator of fetal acid base balance, especially oxygenation of the autonomic nerve centres within the brain, and absent variability is therefore predictive of cerebral asphyxia. A thorough history of each case should be determined prior to CTG interpretation, and instances where variability is persistently reduced without explanation, should be viewed with caution.

Accelerations at this gestation may not be present or may be significantly reduced with a lower amplitude (rise of 10 beats from the baseline rather than 15 beats). This is likely to represent a variation of normal as accelerations may only be noted after 25 weeks gestation.

**Figure 2:**
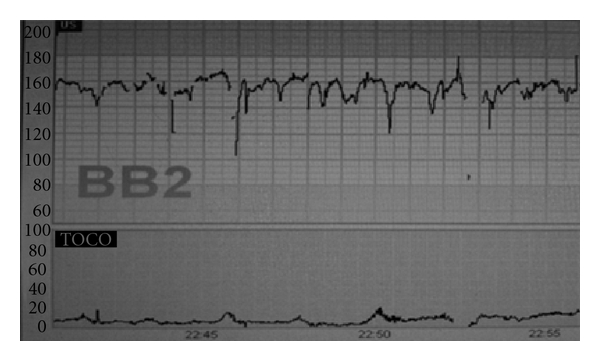
CTG of a fetus at 26 weeks of gestation: note higher baseline heart rate, apparent reduction in baseline variability, and “shallow” variable decelerations.

Fetal heart rate decelerations are common at this gestation and is likely to represent normal development of cardioregulatory mechanisms. In the presence of other reassuring features of the CTG (as outlined above), these decelerations should not be considered as indicative of hypoxia, and interventions should be avoided based on this parameter alone.  Figure 2 shows CTG of a preterm fetus at 26 weeks.

### 4.2. 26–28 Weeks

Within this group, fetal heart rate tracings will show many similarities to the 24*–*26 week gestation cohort. After 27 weeks gestation, the frequency of variable decelerations observed is generally reduced [5]. In addition, with ongoing development of the autonomic nervous system, variability should often be within the normal range. Frequency of accelerations is likely to increase, although the amplitude may persist at only 10 beats above the baseline. Likely, iatrogenic causes of fetal heart rate abnormalities (as mentioned above) should also be noted and documented.

**Practice Points**

Survival in this group is significantly higher than those between 24*–*26 weeks as survival improves approximately 10% every week during this period. Approximately half of those babies who survive may develop long-term neurological or developmental defects. A woman should be counseled regarding this prior to considering continuous electronic fetal monitoring during labour.

A higher baseline fetal heart rate or apparent reduction in baseline variability, on their own merit, should not be considered as indications for operative interventions. Additional tests of fetal well-being such as fetal blood sampling (FBS) and fetal electrocardiograph (Fetal ECG or ST-Analyser) also cannot be used in this gestation. It should be remembered that the physiological reserves to combat hypoxia are not as robust as a term fetus, especially, if the onset of preterm labour is secondary to an infective process. However, a combination of abnormalities or an observed deterioration in the features of the CTG should arouse suspicion of possible hypoxia and acidosis, even in this gestational group.

### 4.3. 28–32 Weeks

With increasing gestation the baseline fetal heart rate is likely to decrease from the upper limits of the normal range. Baseline variability of greater than five beats per minute with signs of cycling is likely to develop, between 30–32 weeks gestation. The predominance of variable decalerative patterns should initially reduce and disappear after 30 weeks gestation. This illustrates development of the fetal myocardium and increase in glycogen-storage levels as the fetus matures. Persistence of late decelerations within this cohort is likely to represent ongoing uteroplacental insufficiency. In this situation, the blood flow within the intervillous space is decreased resulting in accumulation of carbon dioxide and hydrogen ion concentrations. In the noncompromised, nonacidaemic fetus, intermittent hypoxia results in decelerations with subsequent transient fetal hypertension [8]. With passage of time, continuation of this hypoxic insult will lead to acidaemia, loss of initial “compensatory” hypertensive response, and may proceed to cause permanent cerebral injury. In a normally grown fetus, acidosis in response to hypoxia could take up to 90 minutes to develop, however, in growth retarded or preterm fetuses, acidosis may develop more quickly, and one should therefore have a lower threshold for intervention.

**Practice Points**

Survival dramatically increases beyond 28 weeks as the fetal organs are relatively mature and there is significant improvement in fetal neurological development. Hence, fetal monitoring is recommended in this gestational group.

Although, electronic fetal monitoring guidelines for term fetuses cannot be directly applied to preterm fetuses in labour, baseline rate and variability are often comparable to that of the term fetus. Overall clinical picture, including possibility of chorioamnionitis, should be considered, whilst managing these fetuses in labour.

### 4.4. 32–34 Weeks

**Figure 3:**
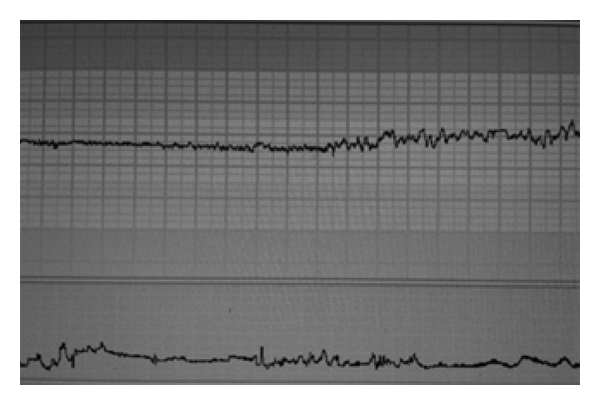
CTG of a fetus at 34 weeks of gestation: note baseline heart rate within the normal range, normal baseline variability with “cycling”.

Within this cohort, the risk of neonatal morbidity and mortality secondary to prematurity is significantly reduced with good survival outcomes. Continuous fetal heart rate monitoring in this group is recommended, following agreement with the patient. Features of CTG classification into nonreassuring and reassuring (as outlined in Table 1) according to NICE guidelines could be considered. This is because physiological maturity of the cardiovascular system and the neural control of the fetal heart rate during this gestational period is similar to that of a term fetus (Figure 3).

**Practice Points**

Baseline fetal heart rate and variability should be comparable to the term fetus and accelerations with an amplitude of greater than 15 beats from the baseline should be present as an indicator of fetal well-being. Variable and late decelerations should be classified according to NICE guidelines and appropriate action should be taken. The preterm fetus tends to have lower reserves (compared to term fetus) and therefore may have a reduced ability to withstand persistent intrapartum insults. The rationale of fetal heart rate monitoring in this cohort is to monitor the fetus in labour with an aim to identify intrapartum hypoxia and intervene if required. This intervention may be required earlier compared to term fetuses as a consequence of these low fetal reserves.

## 5. Role of Additional Tests of Fetal Wellbeing in Monitoring a Preterm Fetus

Several additional tests of fetal well-being are used in labour, which include fetal blood sampling (FBS), fetal pulse oximetry, and fetal electrocardiograph (STAN analysis). These adjuvants to electronic fetal monitoring were introduced to reduce the false-positive rate associated with CTG monitoring [10]. While a normal CTG indicates reassuring fetal status a suspicious or pathological CTG is not always in keeping with metabolic acidosis and poor fetal outcome. The poor-positive predictive value of CTG in addition to variation in CTG interpretation can often lead to unnecessary intervention and high-operative delivery rates [11]. 

### 5.1. Fetal Blood Sampling

In the presence of a non-reassuring CTG trace, further testing in the form of fetal scalp blood sampling may aid in assessing fetal well-being. After rupture of membranes and once the cervix is adequately dilated (>3 cm), sampling a small amount of blood from the fetal scalp can be used to measure pH or lactate and thus detect acidosis. It is not recommended in fetuses with bleeding disorders and is contraindicated in pregnancies complicated with HIV, Hepatitis B or C as it may increase vertical transmission. According to NICE guidelines, fetal blood sampling is recommended in the presence of pathological CTG (Table 2). If the pH value is <7.20, immediate delivery is recommended, whereas a pH of 7.20–7.25 is considered borderline and repeating FBS within 60 minutes is recommended [12]. 

With regards to the pre-term fetus, fetal blood sampling has not been validated in this group. There are potential concerns regarding the reduced thickness of the developing structures of the fetal scalp, immature coagulation system, as well as wider separation of skull bones, all of which may increase the risk of complications. Moreover, studies have shown fetal acidosis to occur more often in pre-term fetuses delivered before 34 weeks than those delivered between 34–36 weeks [5]. Despite this high rate of fetal acidosis, the short-term fetal outcome was good and in subsequent repeat blood-sampling pH values had normalized [5]. This high rate of dramatic fetal acidosis in the preterm may represent an alternative intrapartum compensatory mechanism. Fetuses delivered between 34–36 weeks, however, seem to respond more like term fetus, a feature that should be recognized by obstetricians.

### 5.2. Fetal Pulse Oximetry

Fetal pulse oximetry was first introduced in clinical practice in the 1980s. It provided a means of monitoring fetal oxygen saturation of fetal haemoglobin that is measured optically (similar technology for pulse oximetry in adults) during labour. In non-reassuring CTG traces, pulse oximetry was initially felt to provide a more sophisticated way of detecting adverse neonatal outcome. Several studies defined a critical threshold of <30% SpO2 persisting for greater than ten minutes as a predictor of fetal acidosis and poor neonatal outcome [13]. This cut off value yielded a sensitivity of 81% and specificity of 100% to predict scalp pH of <7.2 [14]. Recent large RCT's, however, have demonstrated no reduction in operative delivery rate or in predicting adverse neonatal outcome [15]. This mode of fetal monitoring now remains obsolete and the manufacturers have ceased production.

### 5.3. Fetal ECG (ST Analyser or STAN)

This technology is based on analyzing the ST segment of the fetal myocardium for ischaemic changes during fetal hypoxia as well as determining the ratio between the T wave and QRS complex (T/QRS Ratio) of the fetal ECG. The latter is altered secondary to release of potassium during glyocogenolysis in the fetal myocardium mediated through that catecholoamine surge, which occurs during hypoxic stress. Myocardium of a preterm fetus has less stored glycogen with increased water content and also the epicardial-endocardial interphase is much smaller than a term fetus. Hence, ST analyser is not recommended prior to 36 weeks of gestation as it may not be reliable due to changes in the myocardial composition described above.

### 5.4. Preterminal Trace

A fetus that demonstrates features of preterminal trace has exhausted all its reserves to combat hypoxia and hence immediate delivery is recommended [16]. However, caution should be exercised in fetuses prior to 28 weeks that demonstrate such features as perinatal outcome is poor in this group. Hence, a woman should be counseled that the risks of operative intervention may outweigh the benefits.

## 6. Conclusion

Continuous electronic fetal monitoring of preterm fetuses poses a clinical dilemma to clinicians caring for these fetuses during labour. Although, clinical evidence-based guidelines and recommendations exist for monitoring term fetuses during labour, there is paucity of scientific evidence in the preterm group. Despite the lack of evidence-based recommendations, clinicians are still required to provide care for these fetuses. Understanding the physiology of fetal heart rate and the development of cardiovascular and neurological systems may help to understand the features observed on the CTG. It is important to realize that physiological reserves available to combat hypoxia are less than those available to a term fetus. Hence, a preterm fetus may suffer a hypoxic insult sooner than its term counterpart. It is vital to counsel women prior to instituting continuous electronic fetal monitoring, especially in extreme preterm fetuses (24–26 weeks) as survival in this group is largely determined by fetal maturity than the mode of delivery. In view of the absence of guidelines and recommendations monitoring preterm fetuses, we have produced a management algorithm “ACUTE” to aid continuous intrapartum fetal monitoring in fetuses prior to 34 weeks (Table 3). Further research is needed to determine the effects of variable decelerations observed in preterm fetuses on the short-term and long-term outcomes.

## Figures and Tables

**Table 1:** Categorizing individual features of CTG according to NICE guidelines.

Feature	Baseline (bpm)	Variability (bpm)	Decelerations	Accelerations
Reassuring Nonreassuring	110–160 100–109 161–180	>5 <5 for 40–90 minutes	None Typical variable decelerations with >50% of contractions for over 90 minutes. Single-prolonged deceleration for up to 3 minutes.	Present The absence of acceleration with otherwise normal trace is of uncertain significance
Abnormal	<100 >180 Sinusoidal pattern >10 minutes	<5 for 90 minutes	Either atypical variable decelerations with >50% of contractions or late decelerations, both for over 30 minutes. Single-prolonged deceleration for more than 3 minutes.	

**Table 2:** Interpretation of fetal blood sample (FBS) results.

FBS result	Interpretation
>7.25	Normal FBS result
7.21–7.24	Borderline FBS result
<7.20	Abnormal FBS result

**Table 3:** Proposed Management Algorithm “ACUTE” for intrapartum fetal monitoring (CTG) in preterm gestations (<34 weeks).

A	Assess survival and long-term outcome at the *given* gestational age.

C	Consider the wider clinical picture:presence of co-existing infection, maternal age, condition of the fetus (severe growth restriction, congenital malformations), wishes of the woman (e.g., request to “do everything possible” in view of IVF conception, previous preterm losses) in formulating management plan.

U	Understand normal fetal cardiovascular and nervous system physiology at the *given* gestation in interpreting the CTG.

T	Treatment of underlying predisposing factors of uterine irritability (infection, antepartum haemorrhage) and treatment of preterm labour (tocolytics and steroids, if appropriate) to optimise maternal and fetal outcome.

E	Evaluate maternal risks of operative interventions (classical C. section, haemorrhage, infections, increased risk of uterine rupture in future pregnancies) and potential fetal benefits (survival and long-term morbidity) due to commencing continuous electronic fetal monitoring at the *given* gestation and counsel appropriately.
